# Basic principles of genetics and genetic counselling

**DOI:** 10.1016/j.ipej.2025.05.002

**Published:** 2025-05-27

**Authors:** Nina J. Beelen, Jitendra Vohra, Job A.J. Verdonschot

**Affiliations:** a Department of Cardiology, Cardiovascular Research Institute Maastricht (CARIM), Maastricht University, Maastricht, the Netherlands; b The Royal Melbourne Hospital, Parkville, Victoria, Australia; c Department of Clinical Genetics, Maastricht University Medical Center, Maastricht, the Netherlands; d European Reference Network for Rare, Low Prevalence and Complex Diseases of the Heart (ERN-GUARD-Heart), Maastricht, the Netherlands

## Abstract

Genetic counselling and testing is a fast growing field in modern medicine. Genetic analysis can aid in the diagnosis, treatment, management and prevention of (genetic) diseases in patients. This review provides an overview of the fundamental principles of genetics from a clinical perspective, focusing on the practical application and interpretation of genetic testing. Key concepts, including genetic expression and inheritance patterns, are discussed to give a better understanding of the subject. There is a large variety of genetic testing methodologies, from single gene testing to whole genome sequencing (WGS) that have their respective advantages, disadvantages and clinical implications. Interpretation of genetic results is performed on different levels: from the molecular level (variant interpretation and classification) to the clinical level (association of genetic variants with disease and phenotype). The correct interpretation of the results is essential to use genetics for clinical decision making. Challenges in the field include, but are not limited to, variants of unknown significance, incidental findings, and the familial forms of disease that remain gene-elusive with the current technologies. Pre- and post-test genetic counselling is essential to ensure that patients and their family members are aware of the options and implications of genetic testing, which will significantly benefit patients with coping. This review is based on the latest European and American guidelines, to give an up-to-date overview of rapid advancements of genetic testing and counselling. A dedicated follow-up article in this series will explore the Indian perspective in greater depth.

## 1 Introduction

Genomics is the study of all of the genes of a person (the genome), including both the coding and non-coding parts of our DNA [1]. Genetics describes the study of heredity, *i.e.,* the function and composition of single genes, which are specific sequences on our DNA that code for functional proteins. The scientific advancements in the past decades have made it possible that analysis of our genome has become an integral part of health care, and are essential in the road towards personalized medicine [2]. Technological improvements have reduced the time that is necessary to sequence the genome, thereby also reducing the cost of genetic sequencing. On the other hand, knowledge on genetic variation and the relation of genetic variants with disease has significantly improved, providing the opportunity to base clinical decision-making on the results of a genetic test [[3], [4], [5]]. Although genetic testing has received a prominent role in all fields of health care (*e.g.,* oncology, cardiology, ophthalmology), future advancements, such as gene therapy and genetic risk profiles, will make the role of genomics and genetics in patient management absolutely essential. The current review provides an overview of the basics of genomics, genetic testing and the interpretation of genetic results, but also provides a clinical practical perspective on how to implement genetic testing in clinical practice.

## 2 DNA, genes and inheritance patterns

A foundational understanding of DNA, chromosomes, and their inheritance patterns is essential for the interpretation of genetic results in a clinical context. Deoxyribonucleic acid (DNA) functions as the molecular blueprint for all living organisms, it encodes all the information required for cellular function [1]. DNA consists of four nucleotide bases: cytosine (C), guanine (G), adenine (A) and thymine (T). The nucleotides are paired via hydrogen bonding in a specific way: C always pairs with G, and A always pairs with T, forming complementary double-strands of DNA. These strands contain specific sequences of nucleotides, also known as genes, that can be transcribed into messenger ribonucleic acid (mRNA), which can then be translated into proteins. DNA molecules are tightly coiled around histone proteins and arranged in thread-like structures called chromosomes. The human genome consists of 23 pairs of chromosomes. Each individual inherits one chromosome from the mother and the other from the father. The 23rd pair determines the biological sex and is also known as the sex chromosomes. Biological females have two X-chromosomes and biological males have one X- and one Y-chromosome. Genetic diseases may be inherited in families according to specific patterns [3,6]. The five primary modes of inheritance are outlined below.

**Fig. 1:**
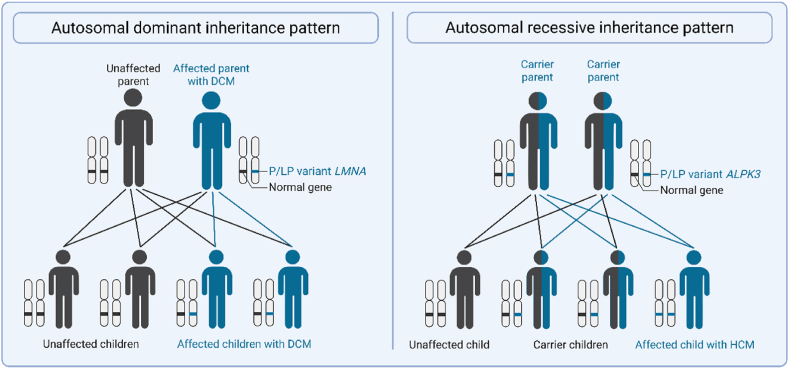
**Autosomal dominant and autosomal recessive inheritance patterns.** Abbreviations as in text.
• Autosomal recessive diseases require pathogenic variants in both alleles of a gene (homozygous) for the phenotype to develop. When only one of the genes has the pathogenic variant the individual usually will not develop the disease (heterozygous), but will be a disease carrier. The affected homozygous individual has a 100 % chance of transmitting an affected gene to their child. This child will then be a disease carrier and not develop the disease, unless the other parent also would be a disease carrier or affected themselves. A heterozygous carrier has a 50 % chance of transmitting the affected gene. If both parents are carriers, there is a 25 % chance that their child will be homozygous and develop the disease. An example of an autosomal recessive disorder is hypertrophic cardiomyopathy caused by P/LP variants in *ALPK3* (Fig. 1).
• X-linked dominant diseases occur when the pathogenic gene variant is located on the X-chromosome. Due to the dominant nature of inheritance, both males and females with the variant will develop the disease. However, fathers will always transmit the disease to their daughters and never to their sons. Mothers have a 50 % chance of transmitting the disease to their children, regardless of the sex of the child. An example of a x-linked dominant disease is Danon disease caused by P/LP variants in *LAMP2* (Fig. 2).

• Autosomal dominant inheritance occurs when a single pathogenic gene variant from one parent is sufficient to cause the disease. In this case, an affected individual has a 50 % chance of transmitting the disease causing gene to their child (*i.e.,* either the gene with the variant or the wild-type gene on the other chromosome will be transmitted). An example of an autosomal dominant disease is dilated cardiomyopathy caused by P/LP variants in *LMNA.* (Fig. 1).

**Fig. 2:**
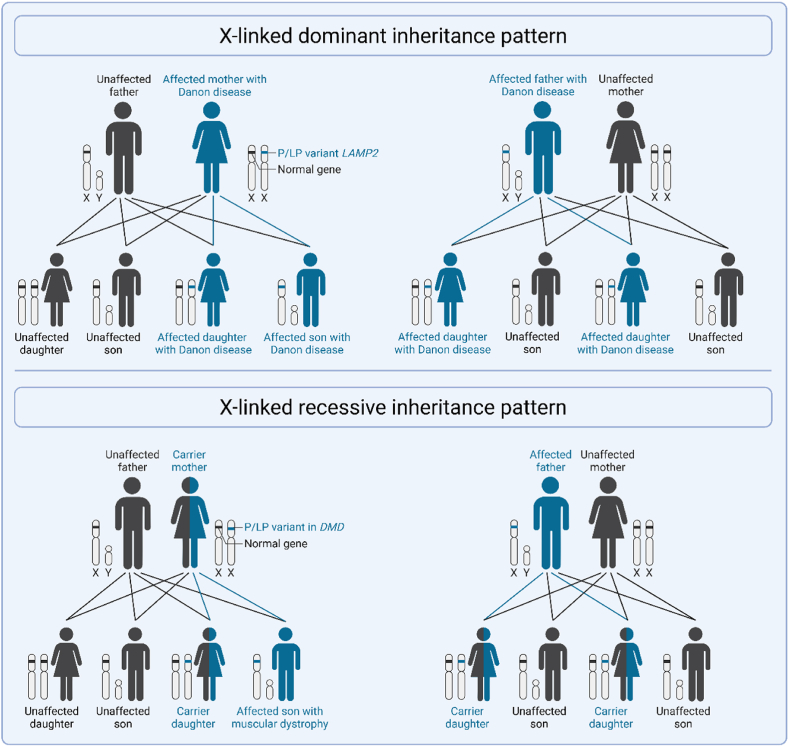
**X-linked dominant and X-linked recessive inheritance patterns.** Abbreviations as in text.
• X-linked recessive diseases primarily affect males. In this case, the pathogenic disease is located on the X-chromosome. Therefore all males, who only have one X-chromosome, will develop the disease. Females, who have two X-chromosomes, can be unaffected or may have a variable expression of the disease, due to their second unaffected X-chromosome. Families with X-linked recessive diseases often only have affected male, but not affected female family members in each generation. An example of a x-linked recessive disease is muscular dystrophy with cardiac involvement caused by P/LP variants in *DMD* (Fig. 2).
• Mitochondrial diseases occur when the affected gene involves the mitochondrial DNA (mtDNA). The mtDNA is different from the nuclear DNA, and contains only 37 genes. Every mitochondria contains multiple copies of mtDNA, meaning that one cell will contain many copies of mtDNA. If a pathogenic variant is located in the mtDNA, it is unusual that every mitochondria contains the pathogenic variant. The burden of affected mtDNA is expressed as a percentage (*i.e.,* heteroplasmy). For example, a heteroplasmy of 34 % indicates that 34 % of the mtDNA carries the pathogenic variant. mtDNA is exclusively transmitted through the maternal line, causing all children to have the disease when the mother has a pathogenic variant in the mtDNA, although the heteroplasmy can vary. Male offspring will also be affected, but they will not pass on their mtDNA to their children. An example of a mitochondrial disease is cardiac involvement in MELAS syndrome caused by *MTTL1* (m.3243A > G) (Fig. 3).

**Fig. 3:**
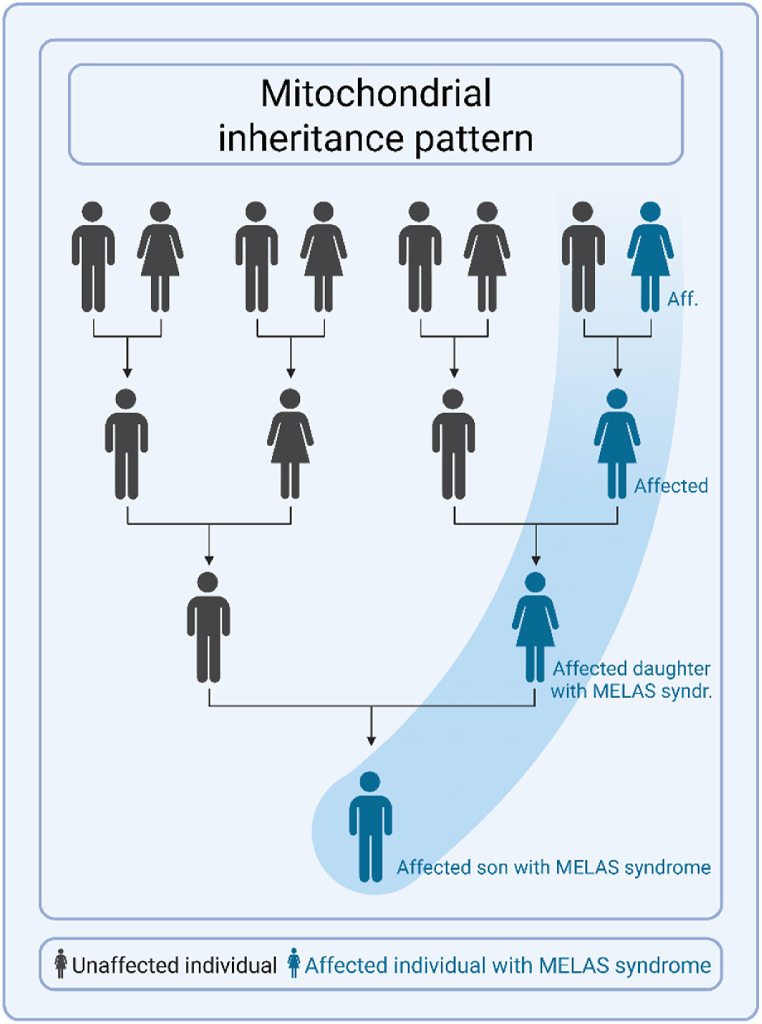
Mitochondrial inheritance pattern.

**Fig. 4:**
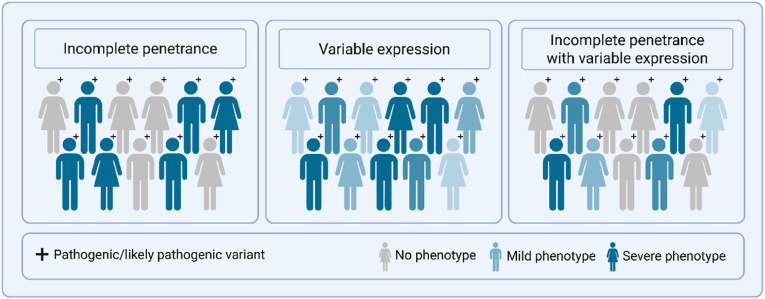
Incomplete penetrance and variable expression.

While these five modes represent the most commonly described inheritance patterns, there are many more modes of inheritance patterns that exist (*e.g.,* digenic, oligogenic inheritance). The majority of inherited cardiac diseases follow an autosomal dominant inheritance pattern. However, not everyone with a pathogenic gene variant will develop the disease, a phenomenon referred to as incomplete penetrance. There can also be great variety in the severity of disease among individuals with the same pathogenic gene variant, even within families, an observation referred to as variable expression. Although the exact factors that can explain the incomplete penetrance and variable expression of genetic diseases remain largely unknown (Fig. 4), the disease will probably develop due to a combination of the individuals genetic predisposition, environmental factors, disease modifiers, and/or co-morbidities [7]. Another important aspect in genetics is the phenomenon of pleiotropy, where a single pathogenic or likely pathogenic variant can cause multiple diseases. For example, variants in the *LMNA* gene can lead to dilated cardiomyopathy, arrhythmias and conduction abnormalities, or even non-cardiac diseases such as progeria and neuropathies [8].

## 3 Methods of genetic testing

Genetic testing is the analysis of the DNA and genes of an individual. The most common and reliable method for identifying a genetic disease is often through analysis of DNA isolated from a peripheral blood sample [9]. DNA from the peripheral blood is isolated from the leukocytes. Alternative sampling methods include saliva swabs, skin or muscle biopsies or amniotic fluid or chorionic villus sampling. Genetic testing can be performed using a range of different methods. The reliability and success of each method is based on the genetic etiology of the disease in question, the clinical context and the availability of prior genetic information [5,[9], [10], [11]]. For example, it is possible that the pathogenic gene variant is limited to a specific cell type, which is called genetic mosaicism. In that case DNA isolated from the blood will not detect the variant, and a sample of the affected tissue will be necessary. There are different approaches to genetic testing (Table 1).

• Single gene testing is performed using fluorescent probes to label the specific nucleotides, allowing for a precise identification of the nucleotide sequence of one gene. It can be used to identify individual exons and their exon-intron boundaries or to analyze the sequence of an entire gene. In a DNA strand, exons are part of the nucleotide sequence that code for specific codons (EXpressed regiONs), while introns are non-coding nucleotide sequences that are removed during the RNA splicing (INTRagenic regiONs). Single gene testing is highly accurate for well-defined monogenetic diseases. However, due to its limited scope, single gene testing may miss diagnoses when the causative gene is unknown, when the disease has a multigenetic etiology, or when multiple genes are associated with a shared phenotype.
• Multigene panels can be performed if more than one gene can be associated with the phenotype of the patient. These panels consist of a curated set of genes that are associated with the disease of interest. When a new gene-disease association is identified for this specific disease, it can be added to the panel. The yield of multigene panel testing is generally higher than the single gene method due to its broader coverage and higher sensitivity for detecting pathogenic variants, however it also increases the likelihood of identifying a variant of unknown significance.
• Whole-exome sequencing (WES) analyses all protein-coding regions (exons) and their adjacent intronic boundaries across the genome. This method is typically performed when the genetic basis of the disease is unknown, or when prior genetic testing (*e.g.*, single gene testing and multigene panel testing) has been inconclusive. As all genes of the human exome can be analyzed, it can also look into genes that are not previously linked to a disease, which aids in the identification of novel disease-associated gene variants. In adult patients, the diagnostic yield of WES is similar to that of a multigene panel, due to the continuous process to update and validate multigene panels [12]. Because of its broad scope, WES carries an increased likelihood of identifying a VUS or incidental (secondary) finding.
• Whole genome sequencing (WGS) is the most extensive approach, which analyses the whole genome of an individual, including the coding, non-coding, and intergenic regions. Compared to WES, WGS can also identify deep intronic variants, and structural variations. However, it shares many of the same challenges with the WES, including the increased chance of identifying incidental (secondary) findings and VUSs. Furthermore, the interpretation and clinical relevance of non-coding variants remain areas of ongoing research.

**Table 1:** **Advantages and limitations per genetic methodology.** A comprehensive overview of the most common and impactful aspects per method. Abbreviations as in text.

	Single gene	Multigene panel	Whole exome sequencing (WES)	Whole genome sequencing (WGS)
Indication	•Phenotype associated with a specific genetic condition •Family history suggest pathogenetic variant in a single gene •Carrier testing for known familial (likely) pathogenic variant	•Phenotype or family history associated with multiple genes (genetic heterogenous) •Syndromes with overlapping clinical features •After negative results single gene testing	•Complex or atypical phenotype •Suspected genetic disorder without a clear single gene candidate •Developmental, metabolic or multisystemic phenotype •Previous genetic testing was inconclusive	•WES analysis was inconclusive •Suspected structural, non-coding regulatory or intronic variants •Case where comprehensive testing is preferred
Advantages	•High sensitivity and specificity for well identified genes •Cost-effective and time-efficient when phenotype-genotype association is clearly defined •Low chance at incidental findings	•Broader diagnostic yield compared to single gene testing •High coverage of phenotype-genotype associations •Cost-efficient and time efficient compared to WES and WGS	•Broad scope, can identify variants across ∼20.000 coding genes •Higher diagnostic yield for heterogenous or heterogeneous phenotypes •Chance at identifying ne genotype-phenotype associations	•Most comprehensive method, can identify coding and non-coding variant structural changes and CVNs •Chance to identify novel disease genes
Limitations	•Limited scope, cannot be used in case of genetic heterogeneity •Large deletions or duplications can be missed •unreliable if phenotype overlaps with multiple syndromes	•Chance at identifying VUS • Test only well defined genotype-phenotype associated genes, cannot detect novel genes • Small chance at identifying incidental findings	• High rate of identifying VUS and incidental findings • Limited to coding regions, cannot identify non-coding or regulatory variants • May not identify structural variants or triplet repeats	• High cost to perform • High chance at identifying VUS and incidental findings • Data interpretation is more complex

Recognizing clinical clues in a patient is still very important in guiding the choice of genetic diagnostics [13]. For example, if the phenotype is very characteristic for a specific disease (*e.g.,* cystic fibrosis), a single gene approach will have the fastest results and minimize unclear findings. However, when a heterogeneous disease such as dilated cardiomyopathy (DCM) presents, it is faster to start with a multigene panel. Some clinical presentations are extremely heterogeneous, and a clinical examination will not provide a diagnosis, such as developmental disorders. In these cases, a broad WES/WGS approach will be the most cost-effective [5].

### 3.1 Genetic variants and classification

Variations in our DNA are common and most are benign, i.e., polymorphisms. However, when a genetic variant is in a gene which is coding for an important protein, it can disrupt its function and lead to disease. Genetic variants can be categorized based on the specific change of DNA and its functional consequences [3,10].

**Fig. 5:**
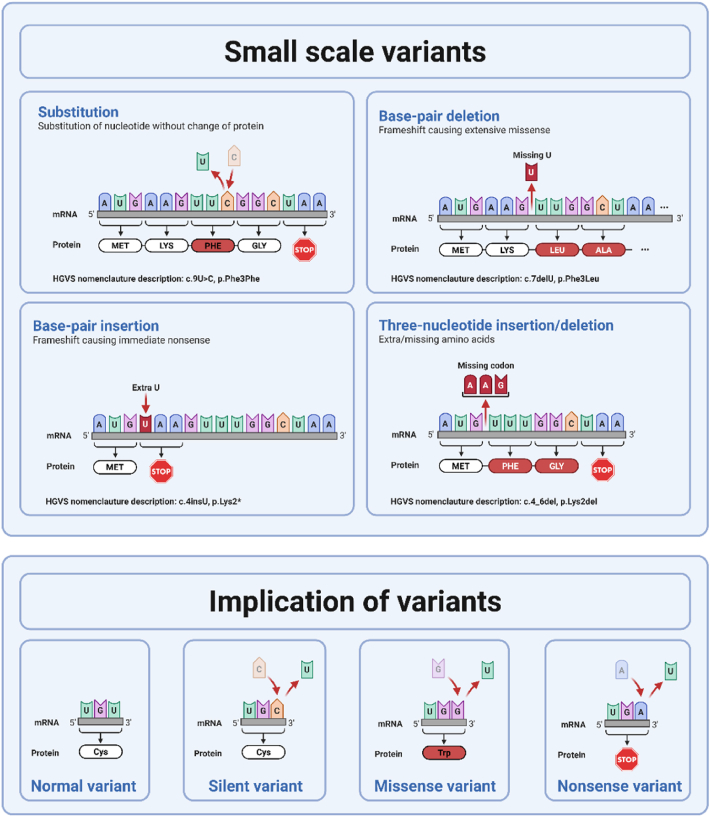
Overview of variant types and their implications.
  o In-frame variants occur when an insertion or deletion takes place in a multiple of three, resulting in a loss or gain of amino acids, potentially altering the function of the protein, but not resulting in a truncated protein (c.2742_2745del, p.Lys915del).
• Splice-site variants affect the process of intron removal during RNA splicing. This may lead to the retention of intronic sequences or exclusion of protein-coding exons, resulting in abnormal transcripts that can impair or disrupt protein synthesis. The exact consequence on the protein is often unknown, if this is not further investigated using RNA sequencing (c.1096-1G > C, p.?).

• Single nucleotide variants (SNVs) occur when a change is made in a single nucleotide. The combination of three nucleotides will translate to an amino acid, and the combination of multiple amino acids will form a protein. A SNV can result in three different manifestations:
  o A silent variant where the changed nucleotide does not alter the amino acid sequence of the protein. For example, changing a codon from GTT to GTA still encodes for the same amino acid, valine (c.30T > A, p.Val10Val).
  o Missense variants occur when a single nucleotide alters the sequence resulting in the coding of a different protein, which can be benign or disease causing depending on its impact on protein function. For example, the change of TTT to TTG will change a phenylalanine to a leucine, thereby changing the amino acids in a protein (c.30T > G, p.Val10Leu).
  o Nonsense variants occur when the nucleotide sequence is changed to form a stop codon, instead of a codon specific amino acid. This results in a shortened protein (*i.e.,* truncating variant) that is most likely non-functional, especially when the change occurs early on in the coding sequence (c.30C > A, p.Tyr10∗). Truncated mRNA can be removed by the cell via nonsense-mediated decay, leading to an insufficient protein as a disease mechanism (*i.e.,* haploinsufficiency), or a truncated non-functioning protein can be built into the cell leading to disturbed cell structure or signaling (*i.e.,* poison peptide).
• Insertions or deletions result in an addition or removal of one or more nucleotides from the DNA sequence. These can be divided into:
  o Frameshift variants, which occur when the number of inserted or deleted nucleotides is not a multitude of three. This causes a shift in the reading frame, causing the sequence to be read one or two nucleotides too early or late, resulting in the synthesis of different amino acids and completely altering the functionality of the protein (Fig. 5). A frameshift variant often results in a truncated variant (c.2742_2749del, p.Lys915Tyrfs∗21).

Variants can also affect large regions of the gene, or even the chromosome. Copy number variations (CNVs) are larger deletions or duplications. The complete gene expression can be upregulated or downregulated based on the type of CNV, altering the gene dosage. Duplications, inversions, deletions and translocations can occur within a single chromosome, but also between two or more chromosomes.

### 3.2 The sequence variant nomenclature system by the human genome variation society

The Human Genome Variation Society is the international standard for the description of genetic sequence variants [4,14]. It aids in the classification of variants and provides a universal language to communicate genetic variation. Since variations in the human genome exist, a reference DNA sequence has been used to compare all new variations to. The letter g. prefix indicates that the variant described has been referenced to the genomic DNA, the letter c. prefix indicates that the variant has been referenced to the coding DNA. The number that is followed by the letter indicates the exact place in the sequence where the variant occurred. To report the exact change in the sequence, it is important to understand what change has been made. All variants should be reported at DNA level. To indicate the exact type of change, characters are used in combination with the abbreviated nucleotides (A, G, T, C). Table 2 provides an overview of the characters used for each variant type. Variants can also be reported on a RNA level, however this should always be additional to the DNA level description. In this case, lowercase letters are used for the nucleotides.

**Table 2:** **Nomenclature reporting genetic variants.** Table of the most common types of variant. For a full overview please refer to HGVS Recommendations for the Description of Sequence Variants14. Abbreviations as in text.

Type of variant	Character	Genomic DNA	Coding DNA	RNA
Substitution	• >	• g.32662262G > A	• c.1318G > A	• r.1318g > u
Deletion	• del	• g.32466684_32466698del	• c.3661 3706del	• r.3661 3706del
Duplication	• dup	• g.32466684_32466698dup	• c.3661 3706dup	• r.3661 3706dup
Insertion	• ins	• g.31792279_31792280insTAGG	• c.7339 7340insTAGG	• r.7339 7340instagg
Inversion	• inv	• g.32481638_32481654inv	• c.3334 3350inv	• r.3334 3350inv
Deletion-insertion	• delins	• g.32867914_32867919delinsTG	• c.112 117delinsTG	• r.112 117delinsug
Translocation	• [ …]	• g.[32841486C > T; 33038273G > C]	• c.[76C > T; 283G > C]	• r.[76c > u; 283g > c]

### 3.3 Classification of genetic variants

Because the term “mutation” is commonly used to describe genetic variation that occurs after conception (*e.g.,* somatic mutations occurring due to external factors as occur in tumors), mutations that are in the DNA from birth (*i.e.,* germline mutations) are referred to as genetic variants that can be classified according to the likelihood of being disease-causing. The classification of genetic variants is standardized by the American College of Medical Genetics and Genomics (ACMG) in collaboration with the Association for Molecular Pathology (AMP) [4]. It categorizes genetic variants based on their predicted effect on the protein and subsequent phenotypic impact.

• Benign variants (B; class 1) are well-established as non-disease causing.
• Likely benign variants (LB; class 2) have strong supporting evidence indicating no association with disease. These variants are often referred to as polymorphisms.
• Variants of unknown significance (VUS; class 3) are subcategorized in a spectrum from “cold” (more likely to be benign) to “hot” (more likely to be pathogenic), however, in most cases the available evidence is either insufficient or conflicting, limiting the ability to determine their clinical relevance.
• Likely pathogenic variants (LP; class 4) are associated with a high probability of disease causation (>95 %), but lack definitive evidence.
• Pathogenic (P; class 5) variants have a robust, well-established evidence demonstrating a direct association with a disease phenotype.

Multiple lines of evidence are required to support the classification of a variant. These include, population frequency data, clinical evidence, family history and co-segregation analysis, results from functional assays and in vitro studies, as well as computational and predictive data. The integration of these type of evidence enables a systematic and reliable approach for the interpretation of variants in both research and clinical settings [4]. While the original ACMG/AMP criteria were very general, efforts are being made to develop disease- and even gene-specific criteria for variant classification. Additionally, as population frequency data is an important criterium, sufficient genetic data from a variety of ethnic groups remains very important. Polymorphisms and genetic variation vary greatly based on ethnicity. Insufficient data on the genetic landscape of an ethnic control group will limit the possibility to classify a variant as P/LP, subsequently leading to less genetic diagnoses and clinical implications [11,15].

## 4 Gene curation and gene-disease associations

After genetic testing, detection of genetic variation and classification of gene variants, a genetic result is available. In order to base clinical decision-making on the results of genetic testing, it is of great importance that the association between the gene in which a P/LP variant is detected has a strong association with the observed disease. The gene-disease association needs to be robust, which requires curation. One of the ClinGen activities, a NIH funded initiative, aims to validate gene-disease relationships (https://clinicalgenome.org). Valid gene-disease associations are important for the following aspects [4,5,11,16,17].

- *Composition of gene panels*: as discussed earlier, genetic testing is often performed using specific multi-gene panels for the observed disease in the patient (*e.g.,* cardiomyopathy panel, breast cancer panel). Genetic laboratories that offer these panels, need to develop and curate the composition of their panels, as these panels ideally would only include genes that have a robust association with the disease.
- *Interpretation of genetic variants in a clinical context*: when a more extensive gene panel is used, or even WGS is performed, there is a risk of detecting a P/LP variant in a gene that has no association with the observed disease of the patient (*e.g.,* the finding of a pathogenic variant in *BRCA1* that is associated with breast cancer in a patient with dilated cardiomyopathy without breast cancer), this is called an incidental finding. Knowledge on the gene-disease association will help the medical expert in estimating the clinical relevance of the genetic result. The absence of an association with the observed variant does not exclude a clinical impact of the incidental finding, as a pathogenic variant in *BRCA1* can still be relevant for a patient and their family. It is recommended to discuss incidental findings in a multidisciplinary team, including genetic experts, to determine further management. Recent studies show that the penetrance of P/LP variants is lower when detected outside the context of the associated disease. However, specific guidelines on the clinical management of incidental findings are lacking due to lack of sufficient data. Some genetic laboratories have an established list of high-impact genes that are routinely screened for P/LP variants during genetic testing for any disorder. The finding of a P/LP variant in a gene not associated with the observed phenotype using this strategy is called a secondary finding. About 36 % of the incidental findings are detected in genes associated with inherited cardiac diseases [18]. The penetrance of cardiac disease is significantly lower compared to when the same variant is detected in a family with cardiac disease [19], however, clear guidelines are lacking. Therefore, findings are often disclosed to the patient with comparable recommendations for cardiological follow-up. Future efforts will be necessary to include the context of the variant discovery towards clinical recommendations for patients and their family. The distinction between incidental and secondary findings is based on the strategy that revealed the P/LP variant, the clinical impact and management are comparable. Given to the possible implications a secondary or incidental finding may have, it is advised to inform the patient of this possibility before testing [20].
- *Reverse phenotyping (genetics first approach)*: the finding of a P/LP variant can be a reason to perform further phenotyping, as the gene-disease association with the observed phenotype does not completely fit. An example is the finding of a truncating variant in *MYBPC3* in a patient with dilated cardiomyopathy (DCM). The association of *MYBPC3* and DCM is limited, thus this finding does probably not explain the phenotype. However, there is a definite association between *MYBPC3* and hypertrophic cardiomyopathy (HCM). This finding might be a reason to reevaluate the phenotyping of the patient, as the observed phenotype could be a ‘burnt-out’ HCM.

The fact that there is a lower technical barrier for broad genetic testing makes it necessary for health professionals to remain critical when interpreting genetic results, since genetic variation is enormous and not every detected variant will be clinically relevant for a patient. Knowledge and awareness of gene-disease association is therefore very important in this era of precision medicine and research.

## 5 Interpretation of genetic results

The interpretation of the genetic results is the most crucial step, as it can have clinical implications for the patient and their family. Interpretation takes place at several levels and steps, and should be performed critically.

- *Molecular interpretation of the variant*: as discussed earlier, the classification of a variant can have different levels of certainty for pathogenicity. In general, P/LP variants are ‘actionable’ variants and can guide clinical decision-making. The finding of a VUS is more challenging, as additional evidence will be necessary to reclassify the variant towards pathogenic or benign [4]. For most diseases, it is not recommended to test family members on the presence of the VUS unless it can help to determine whether the variant was *de novo*, meaning that none of the parents of the patient have the same genetic variant. In the case of a VUS, it is recommended to contact the genetic lab that provided the result to discuss what additional evidence can be generated with segregation in the family and if this would be sufficient to reclassify the variant [4]. Segregation of the VUS means that other family members with a comparable phenotype are tested on the variant: if the VUS segregates with the phenotype in sufficient patients, the variant can be reclassified towards LP.
- *Interpretation of the variant in the context of other clinical variables*: a certain class of variants is phenotypically silent, until combined with an external variable [21]. A well-known example are the drug-induced variants in genes associated with acquired forms of long QT syndrome. These variants do not lead to a phenotype, unless combined with QT-prolonging medication [22].
- *Clinical interpretation of the variant*: a genetic result can have clinical impact when a P/LP variant is detected, and the gene-disease association is verified. Most cardiac diseases can be caused by P/LP variants in different genes (*e.g.,* DCM can be caused by more than 20 different genes), a phenomenon referred to as locus heterogeneity. However, although the final disease is the same, there are differences in natural history and prognosis that are described as genotype-phenotype associations (*i.e.,* the age of onset and disease progression of DCM can differ based on the underlying affected gene) [17].

To support variant interpretation, the *Indian Pacing and Electrophysiology Journal* has initiated a half-yearly report on associations between VUS and strong clinical phenotypes.

## 6 Genetic counselling

Although the practical steps of performing genetic testing is relatively easy, the process of implementing genetic testing in clinical care starts with genetic counselling. The primary purpose of genetic counselling is to help patients and their families understand and adapt to their genetic disorder. In this process it is important to discuss the role of genetic testing, and the potential (clinical) implications of a genetic test result. The exact purpose and role of genetic counselling can differ based on the situation. Therefore, the aspects of counselling will be different with children, or with a healthy individual who has a first degree family member with a P/LP variant [3,5].

### 6.1 Pre-test counselling

Before genetic testing is offered to a patient, it is important to have a detailed family history and pedigree, so the potential implications for the family can be discussed. Education about genetics is important to make sure the patient understands how a possible P/LP variant is inherited in the family. The process and logistics of genetic testing should be discussed, including the possible outcomes (P/LP variant, VUS or no variant detected) with associated implications for clinical care and lifestyle. Depending on the test that will be chosen, the chance of incidental or secondary findings should also be disclosed. Finally, the beliefs and understanding about genetic testing should be explored, and where necessary additional psychosocial support should be provided [23]. It is of great importance that patients can make an informed decision whether they would like genetic testing, and are aware of the clinical implications for themselves and the family.

### 6.2 Post-test counselling

When discussing the results of the genetic test with a patient, several points should always be addressed. If a variant is detected, it should be made clear what the classification of this variant is: pathogenic, likely pathogenic or a VUS, and what the clinical implications are for the patient themselves. If a VUS was detected, it is also recommended to confer the options for gathering additional evidence of the variant [4,16]. Afterwards, the implications for family members should be discussed, and how these family members should be informed about their risk, and can get access to genetic counselling (and potentially subsequent genetic testing). Finally, it should be explored if the patient still has a wish for biological children, and which reproductive options are available for them. Receiving the news that someone has a P/LP variant can be devastating, and can evoke emotions as guilt (*e.g.,* when a patient already has children), fear (*e.g.,* as there is no therapy to target the genetic defect), and uncertainty (*e.g.,* as some P/LP variants might be associated with a poor prognosis). These feelings and the patients understanding should be explored, and where possible psychosocial support can be offered. Sometimes it might be recommended to plan an additional consultation after a couple of weeks, when there has been time to let the new information sink in.

Trained and certified (genetic) health care professionals should pay attention to the needs and wishes of the patient, and implementing genetic counselling is the first step towards shared-decision making for genetic testing.

## 7 Perspective of India

Although this article is primarily based on the clinical practice based on European and American guidelines, we recognize that genetic testing and counselling may vary significantly across regions. A dedicated follow-up article in this series will explore the Indian perspective in greater depth, highlighting current logistics, challenges in access and affordability, cultural and ethical considerations, and the evolving role of clinical genomics in India.

## 8 Conclusion

The rapid advancements of genomic sequencing combined with our increasing understanding of genetic diseases lower the threshold to implement genetic testing in routine clinical care for an expanding number of diseases. It is important to know the basics of genomics, genetic counselling and the clinical context in order to implement genetics in a meaningful way to improve the quality of life for patients and their family.

## Declaration of competing interest

The authors declare that they have no known competing financial interests or personal relationships that could have appeared to influence the work reported in this paper.
